# Supplementary material for: TRPV1 feed-forward sensitisation depends on COX2 upregulation in primary sensory neurons
Source: Sci Rep. 2021 Feb 10;11:3514. doi: 10.1038/s41598-021-82829-6 (PMC7876133; doi:10.1038/s41598-021-82829-6)
Supplement: Supplementary file 1 — Supplementary Information [file 41598_2021_82829_MOESM1_ESM.docx]

**TRPV1 feed-forward sensitisation depends on COX2 upregulation in primary sensory neurons**

Tianci Li^1^, Gaoge Wang^1^, Vivian Chin Chin Hui^1^, Daniel Saad^1^, Joao de Sousa

Valente^2^, Paolo La Montanara^1^, Istvan Nagy^1^

^1^Nociception Group, Section of Anaesthetics, Pain Medicine and Intensive Care,

Department of Surgery and Cancer, Imperial College London, London, United

Kingdom; ^2^ Section of Vascular Biology and Inflammation Section, School of

Cardiovascular Medicine and Sciences, BHF Centre of Research Excellence, King’s

College London, London United Kingdom.

**Supplementary Figure 1.**


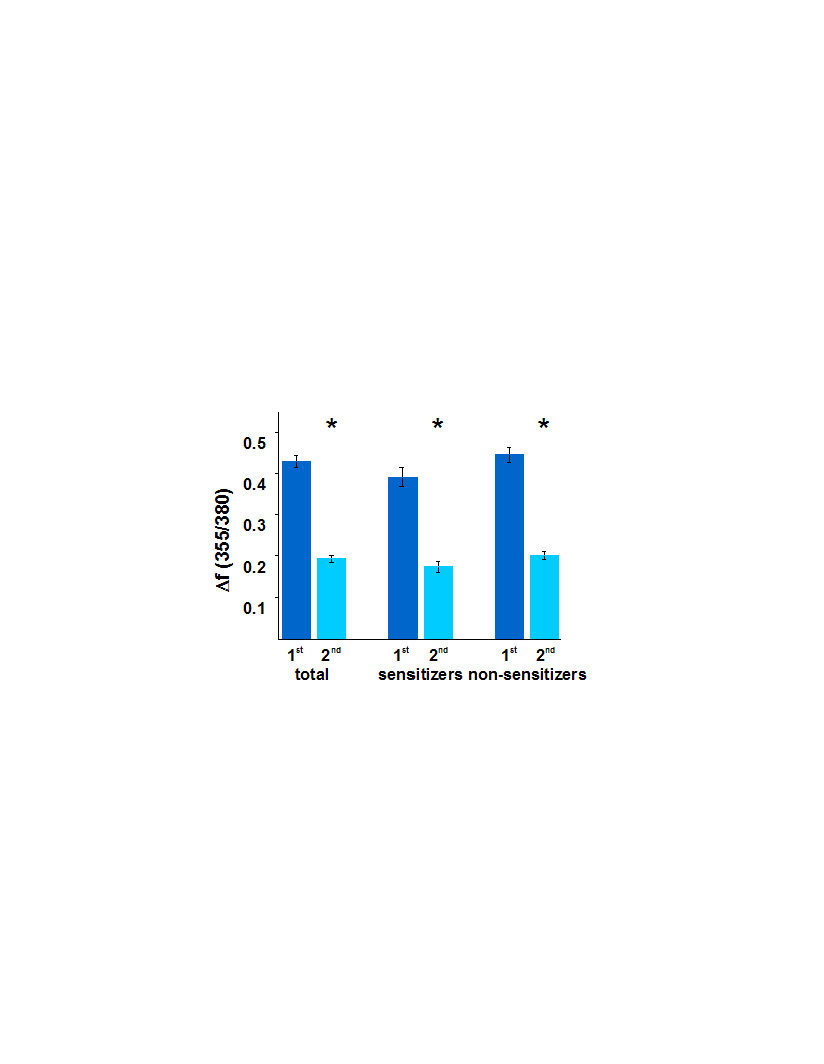


Amplitude of the first (1^st^) and second (2^nd^) KCl-evoked responses in all cultured murine primary sensory neurons (total), in sensitizer neurons (sensitizers) and non-sensitizer neurons (non-sensitizers) in control buffer. The second responses were significantly reduced in all groups (p<0.0001, Student’s t-test, n=288).

**Supplementary Figure 2.**


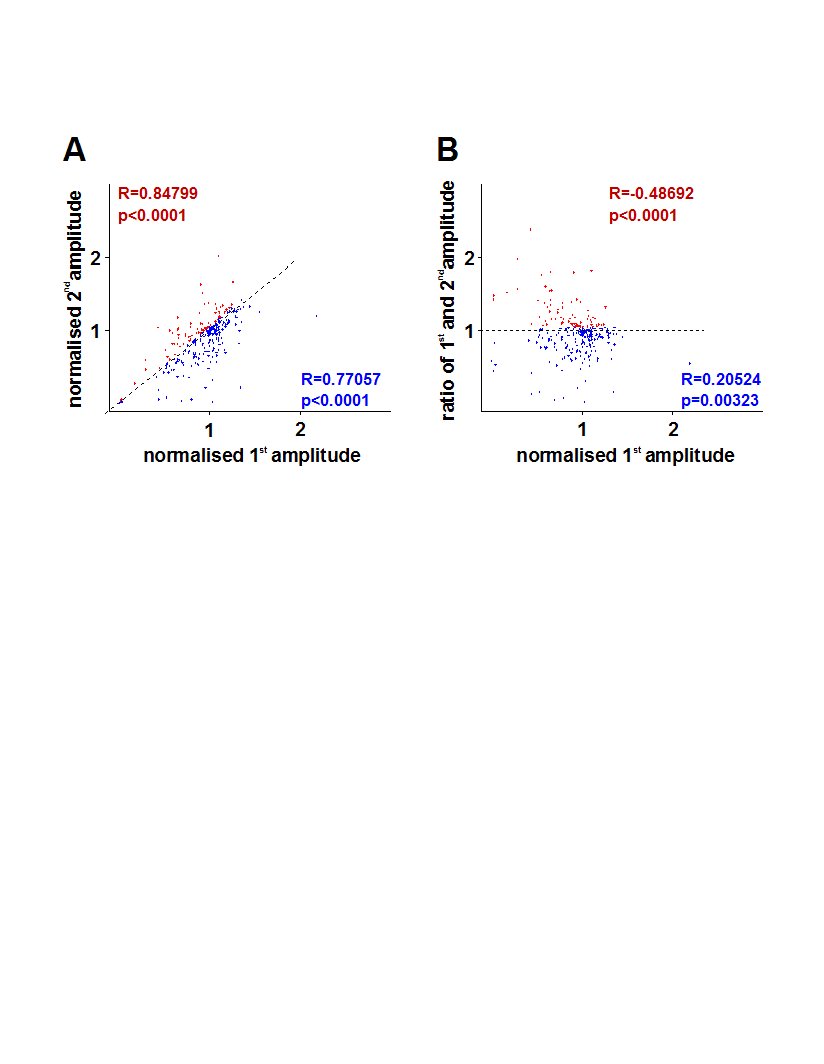


(A) Correlation between normalised capsaicin-evoked first and second responses of sensitizer (red) and non-sensitizer (blue) cells. In both group there is a significant correlation. Dotter line indicates first normalised response/second normalised response=1.

(B) Correlation between the first normalised capsaicin-evoked response and the ratio of the first and second capsaicin-evoked responses in sensitizer (red) and non sensitizer (blue) cells. Sensitizers with smaller first responses tend to exhibit greater increase in the second response. Dotted line indicates a ratio of the first and second capsaicin-evoked responses=1.

**Supplementary Figure 3**


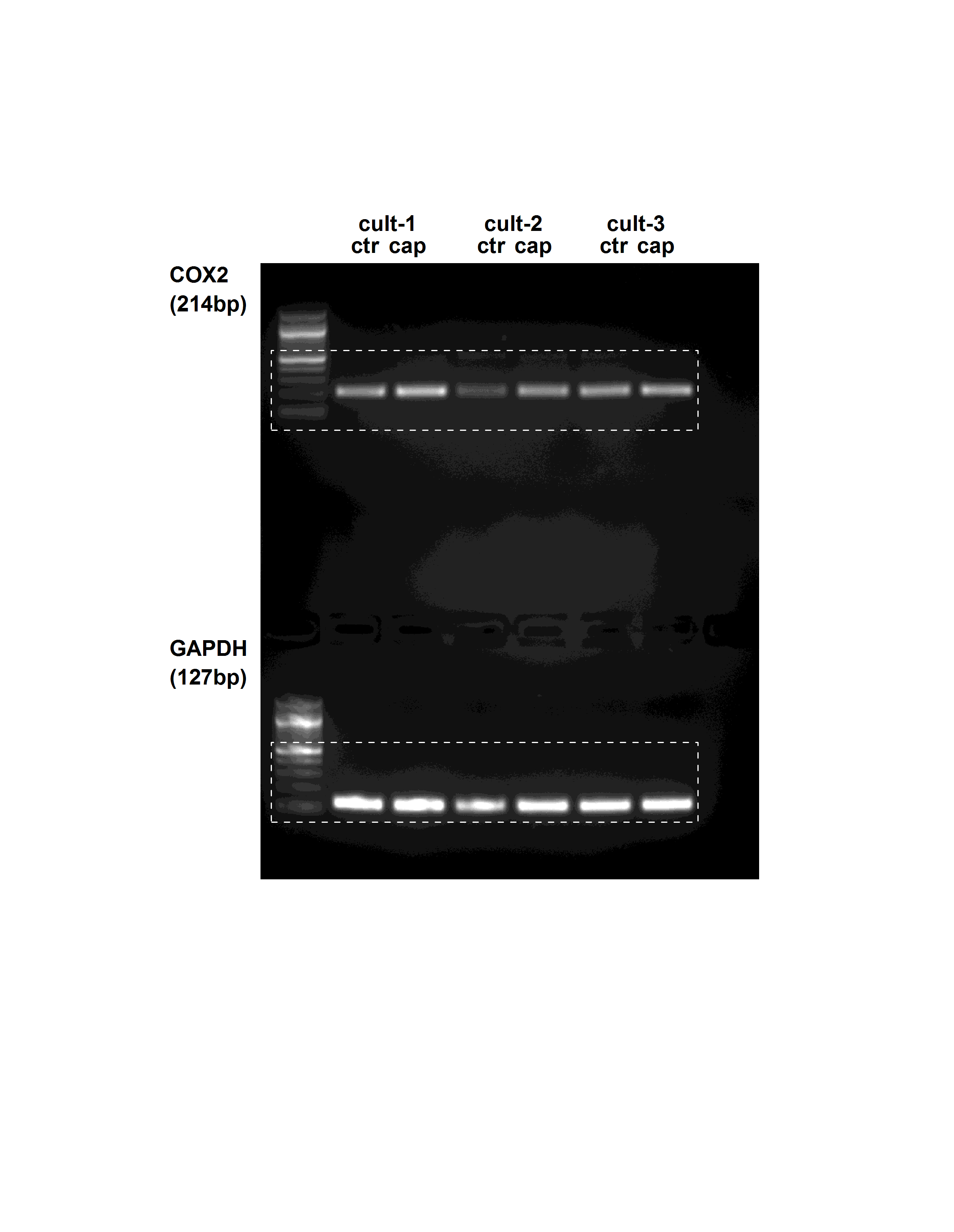


Full gel image of RT-PCR products amplified using primer pairs for murine *ptgs2* (COX2) and *gapdh* (GAPDH) and cDNA prepared using RNA isolated from three cultures (cult-1 – cult-3) of murine primary sensory neurons 25 minutes after incubating the cells in control (ctr) buffer or in the presence of 500nM capsaicin (cap) for 5 minutes. Boxed areas indicate cropped images shown in Figure 2.

**Supplementary Figure 4.**


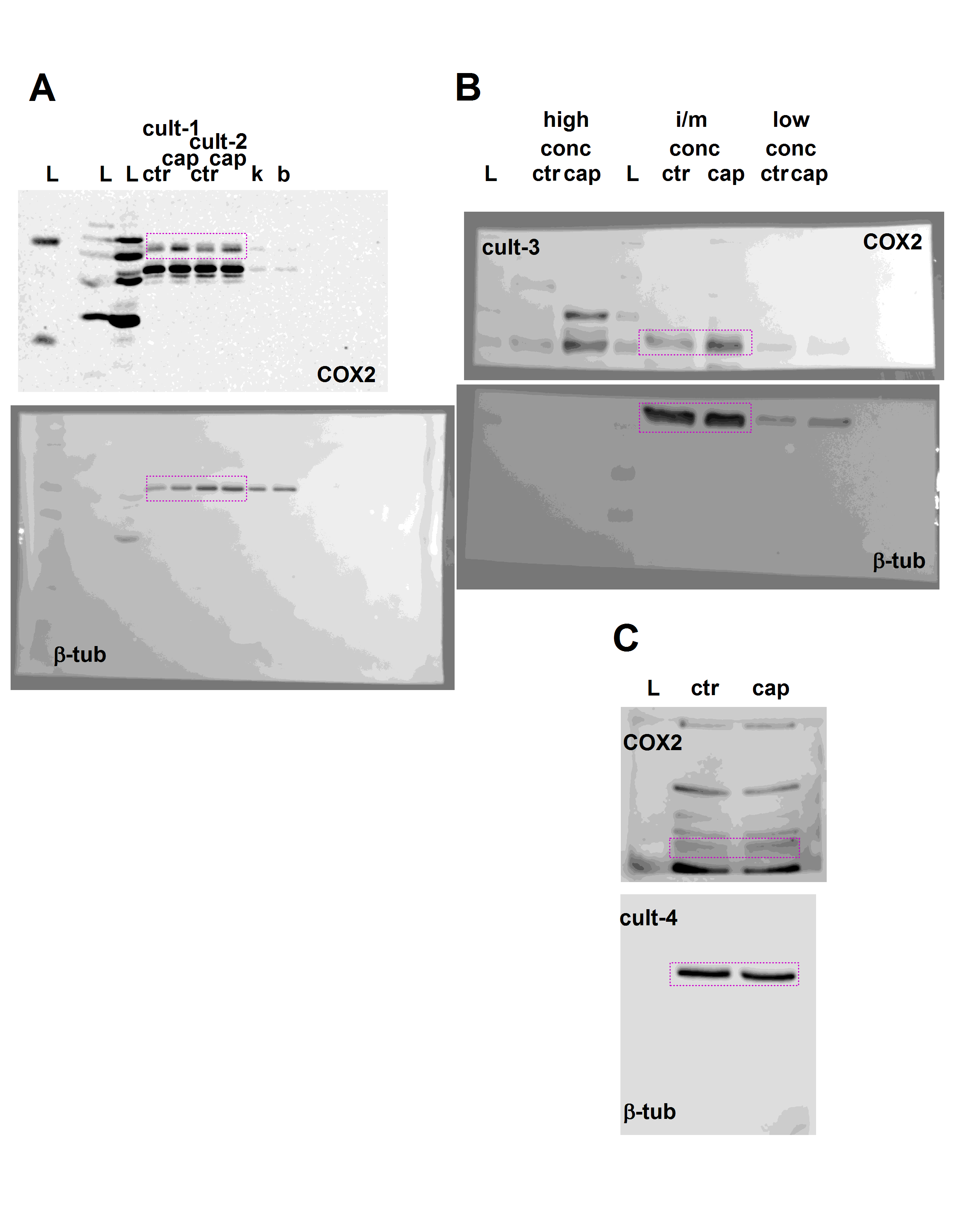


Full gel images of immunoblots used for analysis. Cultures (Cult1-4) were prepared from murine dorsal root ganglia. Proteins were extracted 25 minutes after incubating the cells in control buffer (ctr) or in the presence of 500nM capsaicin (caps) for 5 minutes. Membranes were incubated in antibodies raised against COX2 and β-tubulin as described in methods and indicated in the figure. L indicates size marker. Boxed areas indicate blots used for analysis and cropped images shown in Figure 2. (A) shows images of blots in samples prepared from culture 1 (cult-1) and culture 2 (cult-2). k indicates sample prepared from kidney, while b indicates sample prepared from brain. (B) shows blots in samples prepared from culture 3 (cult-3). In this experiment, the sample was loaded in 3 concentrations (high, intermediate (i/m) and low). (C) shows blots in samples prepared from culture 4 (cult-4).
